# Materials dataset from microgravity levitation experiments on the China Space Station

**DOI:** 10.1038/s41597-025-06428-0

**Published:** 2026-01-16

**Authors:** Yanan Liu, Shengyang Li, Bo Yang, Yunfei Liu, Yunziwei Deng, Jianding Yu, Xuzhi Li

**Affiliations:** 1 Technology and Engineering Center for Space Utilization, Chinese Academy of Sciences, Beijing, 100094 China; 2 Key Laboratory of Space Utilization, Chinese Academy of Sciences, Beijing, 100094 China; 3 University of Chinese Academy of Sciences, Beijing, 100049 China; 4 Shanghai Institute of Ceramics, Chinese Academy of Sciences, Shanghai, 200050 China

**Keywords:** Condensed-matter physics, Characterization and analytical techniques, Thermodynamics

## Abstract

The China Space Station provides new opportunities for containerless materials research through its electrostatic levitation platform, enabling non-contact processing and property measurements of high-temperature materials in microgravity. This dataset presents experimental records from containerless electrostatic levitation experiments conducted both in orbit and through ground-matched experiments under matched configurations. The on-orbit facility integrates tri-axial electrostatic levitation, laser heating, infrared pyrometry, and high-speed diagnostics to support precise observation of materials during high-temperature processes. A ground-matched system with equivalent setup was developed to enable direct comparison between gravitational environments. The dataset contains 565 experimental records, including 420 on-orbit and 145 ground-matched experiments, covering metallic alloys, ceramics, and other material types. It provides key thermophysical parameters, including liquid-phase density and thermal response indicators, enabling systematic investigation of gravity-related influences on material behavior. This dataset offers a valuable resource for research in containerless materials processing, gravitational effect studies, and the development of space-based experimental techniques.

## Background & Summary

Thermophysical properties of high-temperature materials including density, specific heat capacity, viscosity, and surface tension are fundamental to understanding material behavior under extreme conditions^1–4^. These parameters serve as critical inputs for material design, performance prediction, and process optimization. However, under terrestrial conditions, gravity-induced natural convection, thermal gradients, and container interactions introduce significant systematic errors, making it difficult to accurately measure intrinsic properties of materials in their molten state^5,6^.

**Fig. 1:**
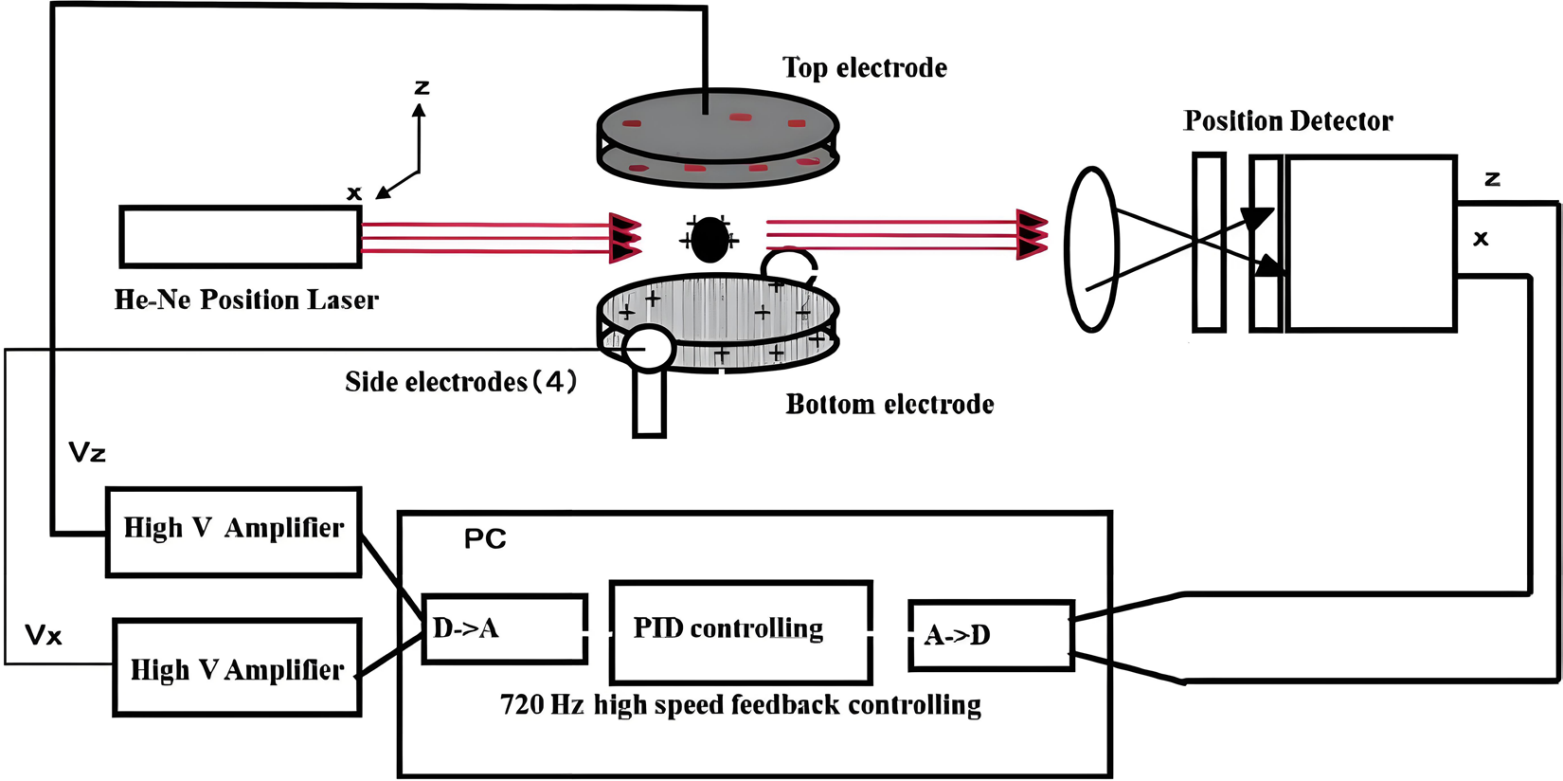
Schematic of the sample position control system (adapted from Ishikawa *et al*., *Journal of the Japanese Society for Microgravity Application*, 2001)^26^.

To overcome these limitations, the China Space Station (CSS) has deployed a containerless materials science experiment rack that integrates tri-axial six-electrode electrostatic levitation (EL), laser heating, infrared pyrometry, and high-speed imaging. This system enables stable suspension and non-contact thermal processing of millimeter-sized samples under high-vacuum and microgravity conditions, significantly reducing measurement uncertainty^7^. Figure 1 shows a schematic of the EL system onboard the CSS, illustrating its core components and feedback control architecture, as conceptualized in the original JAXA design^8^.

Since 2020, Chinese research teams have conducted systematic containerless experiments in space, targeting a wide variety of material classes such as refractory alloys, functional metallic systems, high-performance bio-glasses, and proto-solar nebula analogs. These studies have led to precise measurements of thermophysical parameters under extreme conditions and revealed new behaviors associated with solidification, phase transformation, and interface evolution in microgravity. Such findings have advanced the theoretical foundation of space materials science while also providing valuable insights for optimizing terrestrial processes and designing advanced materials.

Containerless processing in space leverages the unique advantages of the microgravity environment, where non-contact levitation and melting eliminate convection and contamination^9–11^. This approach is particularly valuable in the development of new functional and structural materials, as well as in interdisciplinary research spanning planetary science and cosmochemistry^12^. Compared to earlier platforms such as ESA’s electromagnetic levitation furnace (EML) and JAXA’s electrostatic levitation furnace (ELF), which are optimized for conductive metals and non-conductive oxides respectively, the CSS containerless rack overcomes these material limitations and supports a broader range of systems, including metals, oxides, glasses, and semiconductors^13,14^.

Previous containerless experiments performed with JAXA’s ELF on board the International Space Station (ISS) have demonstrated that microgravity electrostatic levitation enables high-precision thermophysical measurements. Systematic evaluations following the GUM framework have reported typical uncertainties of approximately 1–2% for density, 1–6% for surface tension, and 4–10% for viscosity in metallic melts, with larger values observed for highly viscous oxides^15^. These results have established ISS-ELF as the international benchmark for containerless thermophysical measurements. Against this backdrop, the CSS-based ESL platform presented here achieves comparable uncertainty levels (≤2% for density and ≤10% for viscosity and surface tension), ensuring direct comparability with established ISS-ELF results and underscoring the international competitiveness of the dataset.

To support comparative studies and ensure the reliability of on-orbit measurements, a ground-matched EL system with functionally equivalent architecture to the on-orbit setup was established^16^. This platform enables parallel experiments under controlled vacuum conditions, using identical sample preparation, laser heating, infrared temperature measurement, and data acquisition protocols^17–19^. By synchronizing experimental configurations across gravity environments, the ground-matched experiments offer a valuable reference for interpreting microgravity results, allowing systematic evaluation of gravitational influences on thermophysical behaviors such as volatility, thermal response, and phase evolution^20,21^. This dual-environment design not only enhances the credibility of space data but also establishes a foundation for validating models and extracting gravity-dependent material phenomena^22,23^.

The dataset presented here compiles a total of 565 containerless experiment records, including 420 from on-orbit samples onboard the CSS and 145 from matched ground experiments. The data encompass various sample types—metals, alloys, and ceramics—and are archived in structured CSV format using standardized SI units and annotated fields. Each record includes project metadata, sample descriptors, physical and chemical characteristics, electromagnetic behavior, and extracted thermophysical properties.

The dataset will continue to grow with the return and analysis of post-mission samples, providing new insights into microstructural evolution and composition-phase-performance relationships. The evolving dataset is designed to support a wide range of applications including numerical model calibration, cross-environmental behavior analysis, and AI-based property prediction for containerless processing.

The CSS provides a long-duration, stable microgravity environment for advanced materials research. A key onboard facility, the Containerless Materials Processing Rack (CMPR), utilizes EL, laser heating, and optical diagnostics to conduct container-free experiments under high vacuum. EL eliminates container-induced contamination and suppresses convection and sedimentation, allowing accurate measurement of intrinsic thermophysical properties during melting, solidification, and phase transitions. This technique is particularly suitable for volatile, reactive, and non-conductive materials, for which electromagnetic or aerodynamic levitation techniques are often limited.

## Methods

### on-orbit experimental apparatus and data acquisition

All containerless experiments were conducted using the CMPR onboard the CSS. The system integrates EL, laser heating, real-time optical diagnostics, and synchronized data acquisition to enable container-free thermophysical measurements under high-vacuum conditions.

**Fig. 2:**
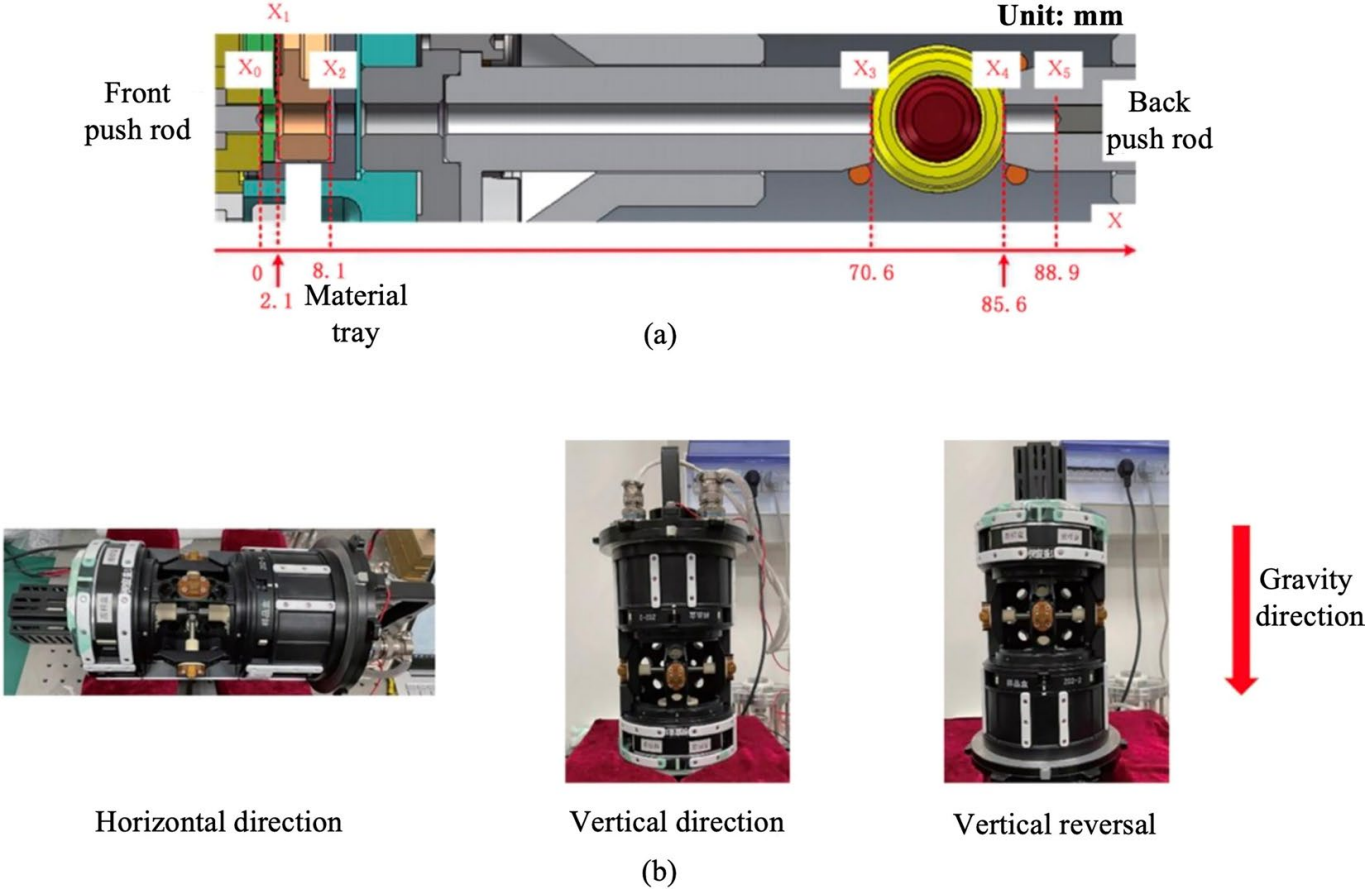
(**a**) Operation structure diagram of push rod in shaft mechanism. (**b**) Orientation of the shaft mechanism with respect to the gravity direction, including three representative configurations: horizontal (left), vertical (middle), and inverted (right).

Experiment sample transfer into the levitation zone is achieved via a motor-driven shaft mechanism, which pushes individual specimens from a sealed sample cartridge into the central electrode region. The push-rod structure and operational layout of the shaft mechanism are illustrated in Fig. 2a. To accommodate variations in orbital attitude, the shaft mechanism supports flexible installation in multiple orientations—including horizontal, vertical, and inverted configurations—as shown in Fig. 2b.

**Fig. 3:**
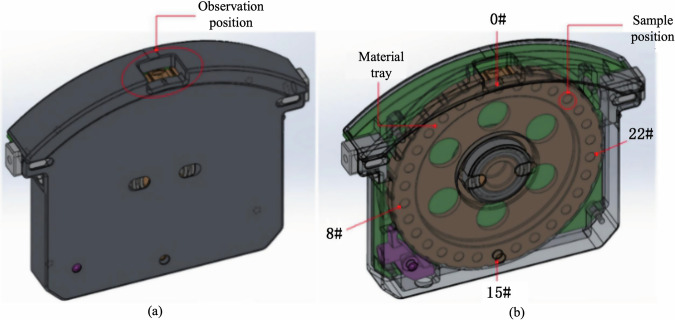
(**a**) Cartridge with integrated optical observation window for confirming sample positioning prior to experiment; (**b**) Rotary tray capable of storing up to 29 spherical specimens in predefined slots (e.g., 0#, 8#, 15#, 22#) for automated delivery to the levitation zone.

The sample cartridge itself contains a rotary tray capable of storing up to 29 spherical specimens. Individual samples are selected from pre-defined slots (e.g., 0#, 8#, 15#, 22#) and automatically delivered to the levitation zone. An integrated optical window allows visual confirmation of sample positioning before experiment execution. The structural layout of the cartridge and rotary tray is shown in Fig. 3.

The levitation subsystem uses a three-axis, six-electrode configuration to maintain non-contact suspension and active position control of the sample. Before levitation, the chamber is evacuated to ~10^−3^ Pa using vacuum pumps. During heating and cooling, electrode voltages are dynamically adjusted in real time to maintain sample stability.

Heating is achieved via multiple laser sources whose beams are directed through optical ports and focused onto the sample surface. Laser parameters such as power and spot size are adjusted depending on the material type and experimental goals.

Temperature is measured using a non-contact infrared pyrometer aligned with the equatorial plane of the levitated sample. Emissivity values are pre-calibrated on the ground and applied based on material type. The thermal history is continuously logged throughout each cycle.

To capture dynamic behaviors such as oscillations and shape changes, imaging techniques are employed to record the sample’s deformation during processing. These visual data are analyzed using axisymmetric oscillation models to derive surface tension and viscosity. The imaging parameters were adjusted based on the sample’s brightness and emissive properties. High-speed imaging was performed at 400 frames per second with a detector resolution of 512 × 512 pixels, an exposure time of 500 μs, and a calibrated pixel size of 0.0166 mm per pixel. The sample mass was measured using a precision balance (±0.01 mg), and an evaporation correction (≤0.5% of the initial mass) was applied by comparing pre- and post-experiment mass. Sample contours were extracted using a Canny edge detector combined with sub-pixel spline fitting, and the projected radial profiles were approximated by polynomial functions. To determine the appropriate order, synthetic spherical contours and glass beads with known diameters were analyzed using 4th-, 5th-, and 6th-order polynomials. The 5th-order polynomial yielded the smallest residuals without systematic bias, and was therefore adopted in this study for volume reconstruction under the assumption of axial symmetry. This workflow enabled robust contour reconstruction and yielded a relative volume-measurement uncertainty of approximately 1.7%.

A central control system synchronizes data streams from all subsystems, including laser heating, levitation voltages, video capture, and temperature logging. Time-series data—such as temperature, laser power, and levitation voltages—are recorded with unified time stamps and stored in CSV format using standardized metadata fields.

Each space-based experiment is paired with a corresponding ground-matched validation trial performed by the respective principal research institution, using similar levitation platforms and measurement protocols. These matched datasets enable consistency checks and systematic analysis of gravity-dependent phenomena.

### Ground-matched experiments

Ground-matched experiments serve as a critical component of the space materials research system, aiming to provide benchmark references and physical interpretations for on-orbit results. To simulate the electromagnetic environment and experimental procedures of the CMPR system onboard the CSS, equivalent ground-matched EL platforms were employed. These platforms are equipped with a vacuum chamber (minimum pressure of 10^−3^ Pa), a three-axis six-electrode EL system, a CO_2_ laser with a wavelength of 1064 nm and a rated power of 30 W, a semiconductor laser with a wavelength of 915 nm and a rated power of 400 W, infrared pyrometry, a high-speed dual-camera imaging system, and multi-channel data acquisition modules. Auxiliary equipment includes a deuterium lamp (for charge excitation), high-voltage amplifiers (±3 kV for horizontal and ±30 kV for vertical control), and a high-precision balance (resolution 0.01 mg). During each experiment, the sample is suspended and heated under vacuum, while real-time adjustments of electric fields, voltages, and laser power enable synchronized acquisition of temperature, image, and electrical signal data.

The testing process covers six key measurement categories: (1) Sample volatility—evaluated by comparing pre- and post-heating mass to assess evaporation loss under high-temperature vacuum conditions; (2) Laser absorption characteristics—analyzed by examining the relationship between laser power and heating rate; (3) Surface morphology after melting and solidification—observed for structural integrity and the evolution of defects; (4) Charging behavior—quantified under deuterium lamp excitation to assess charging efficiency and stability in the electrostatic field; (5) Heating and cooling dynamics—temperature-time profiles are recorded to extract thermal response rates; (6) Thermophysical properties—including density, resonant frequency, and derived viscosity and surface tension. Depending on the material properties and research objectives, a tailored combination of these measurements is selected for each sample.

All experimental data are organized in a standardized format and recorded in the materials database, including basic physical properties, experimental configuration parameters, and thermophysical measurement results. These ground-matched experiments provide comparative references for on-orbit tests, support refinement of thermophysical models, optimization of experimental protocols, and cross-comparison among multiple material systems.

### Data processing

To derive key thermophysical properties from containerless experiments conducted under microgravity, high-speed image sequences and thermal measurements were systematically post-processed. The sample density $\rho$ was calculated using a corrected relation to account for mass loss during evaporation:

1 $$\rho (t)=\frac{m(t)}{V(t)}$$

where $m(t)$ is the effective sample mass corrected using the difference between pre- and post-experiment measurements, and $V(t)$ is the instantaneous volume reconstructed from the 2D projected contour. In our experiments, mass loss during heating was generally ≤0.5%, for which the evaporation-corrected mass $m(t)$ provides a reliable basis for density determination. Assuming axial symmetry, the sample volume was obtained by numerically integrating the squared radial profile extracted from each image frame:

2 $$V=\pi {\int }_{0}^{{R}_{y}}{r}^{2}(y){dy}$$

The radial profile $r(y)$ was fitted to a polynomial function:

3 $$r(y)=\mathop{\sum }\limits_{i=0}^{n}{a}_{i}{y}^{i}$$

where the coefficients ${a}_{i}$ were estimated from image data and $n$ denotes the polynomial order (typically 4–6). Uncertainties associated with balance measurement precision (±0.01 mg) and the applied mass-loss correction were systematically propagated into the density determination using standard error analysis.

Surface tension (γ) was extracted from the dominant frequency of the sample’s shape oscillation using the Rayleigh model^24^ for the quadrupole mode of a free droplet:

4 $$\begin{array}{c}{\rm{f}}=\frac{1}{2{\rm{\pi }}}\sqrt{\frac{8{\rm{\gamma }}}{3{\rm{\rho }}{{\rm{R}}}^{3}}}\Rightarrow {\rm{\gamma }}=\frac{3{\rm{\rho }}{{\rm{R}}}^{3}{(2{\rm{\pi }}{\rm{f}})}^{2}}{8}\end{array}$$

Here, *f* is the oscillation frequency, *R* is the mean radius of the sample during the liquid state, and *ρ* is the measured density. The damping behavior of the droplet was modeled as a decaying harmonic oscillation:

5 $$\begin{array}{c}\begin{array}{c}\Delta (t)={A}_{0}{e}^{-\beta t}\cos \left(2\pi {ft}+\phi \right)\end{array}\end{array}$$

where A_0_ is the initial amplitude, β is the damping coefficient, and ϕ is the initial phase. The damping factor was further converted into a frequency domain quantity:

6 $$\begin{array}{c}\delta =\frac{\beta }{\pi }\end{array}$$

and used to calculate the dynamic viscosity η through the Lamb model for viscous damping of oscillating spheres:

7 $$\begin{array}{c}\eta =\frac{\rho {R}^{2}\delta }{5}\end{array}$$

Oscillation signals were extracted from time series of shape descriptors (e.g., eccentricity or diameter), followed by Fast Fourier Transform (FFT) to identify the dominant frequency and its spectral half-width $\delta$. All derived parameters were aligned with metadata such as sample ID, composition, heating conditions, and temperature profiles to construct a comprehensive thermophysical property dataset. This approach ensures accurate, self-consistent estimation of molten material behavior in microgravity, supporting quantitative material analysis across gravitational environments.

**Fig. 4:**
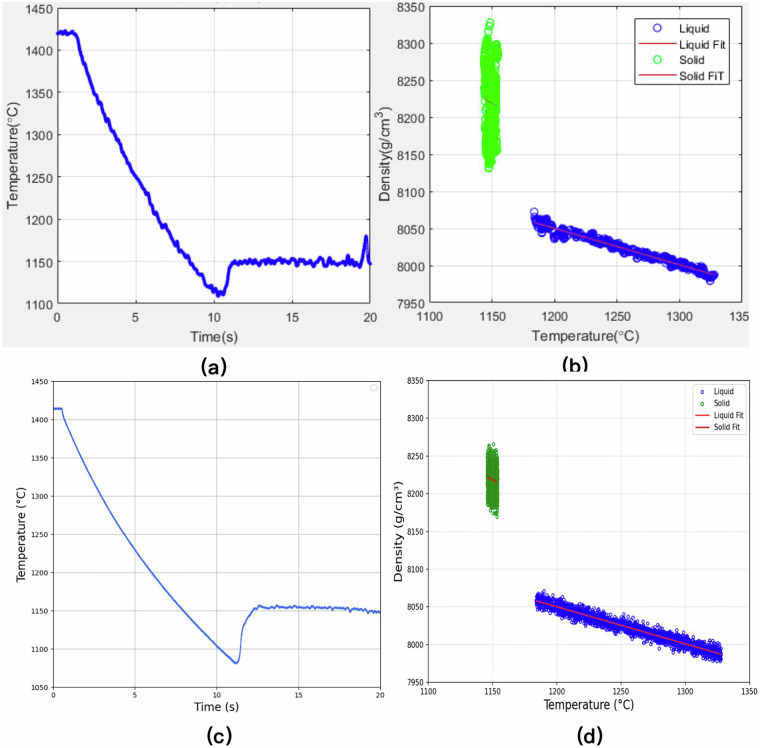
(**a**) Time-temperature curve obtained from the ground-matched experiment. (**b**) Temperature-density curve obtained from the ground-matched experiment. (**c**) Time-temperature curve obtained from the on-orbit experiment. (**d**) Temperature-density curve obtained from the on-orbit experiment.

To further illustrate the thermophysical property extraction process and demonstrate the consistency between ground-matched and on-orbit measurements, Fig. 4 presents representative results for the Ni_60_Nb_40_ sample.

These visualized results confirm that the two experimental platforms—though operating under different gravitational environments—share consistent measurement protocols and data processing pipelines. The comparison provides valuable insight into the influence of gravity on thermophysical behavior, and supports the robustness, interpretability, and future expandability of the dataset.

### Liquid phase density comparison

To further demonstrate the comparative value of this dataset in analyzing matched experiments under different gravitational environments, we conducted a preliminary comparison of liquid phase density data for three representative material systems: Ti-Fe hollow spheres, ZrNbSi alloy, and NiNb alloy. All density values were derived by combining image-reconstructed droplet volumes with mass-loss-corrected effective mass $m(t)$, ensuring consistency with the processing pipeline described in the Data processing section.

For the Ti-Fe hollow spheres, the measured melting point densities under microgravity conditions range from 4.75687 to 4.92198 g/cm³, while the corresponding ground-matched value is 4.88298 g/cm^3^. Although the ranges partially overlap, the on-orbit data display slightly greater dispersion. This variation may be attributed to subtle differences in cavity structures or geometric distortions caused by gravity, which can influence contour reconstruction based on imaging. In contrast, the microgravity environment suppresses convection, sedimentation, and deformation, enabling the molten samples to maintain a nearly ideal spherical shape, thereby improving the accuracy of volume and density measurements.

The ZrNbSi alloy exhibits a more systematic offset between conditions. on-orbit densities are concentrated between 6.67 and 6.86 g/cm^3^, while ground-matched measurements fall between 6.14 and 6.32 g/cm^3^. The higher and more stable values obtained in orbit suggest reduced influence from gravitationally driven surface deformation and buoyancy effects. This is particularly relevant for viscous melts, which are more susceptible to shape distortion under terrestrial conditions, potentially leading to slight overestimation of volume and underestimation of density.

The NiNb alloy includes the most abundant samples in the dataset. The on-orbit density measurements range from 8.08 to 8.19 g/cm³, while the ground-matched values cluster between 7.94 and 8.02 g/cm³. Although the deviation is relatively small, the on-orbit results consistently show slightly higher values and narrower variance. Given the inherently high surface tension and shape stability of the NiNb melt, the microgravity environment further reduces oscillation amplitude and contour uncertainty, enabling more reliable volume reconstruction and density inference.

In summary, all three material systems exhibit a general trend of higher and more stable liquid phase densities under microgravity compared to terrestrial measurements. This indicates not only systematic bias due to gravity-related deformation and convective effects, but also improved contour fidelity and measurement consistency in microgravity. Future work will further incorporate time-resolved contour sequences, modal analysis, and fitting residuals to quantify how gravity-induced shape perturbations affect the uncertainty of density extraction. These efforts will contribute to a more robust assessment framework for thermophysical property measurements in containerless processing.

## Data Records

The dataset is available at 10.5281/zenodo.17156569, comprising 565 containerless levitation experiment records, including 420 on-orbit measurements conducted onboard the CSS and 145 ground-matched matched experiments^25^. All data are provided in standardized CSV format with SI-compliant units and consistent field naming conventions, ensuring broad interoperability, reproducibility, and reusability across research tasks. Each record is conducted “On-Orbit” or as a “Ground-Matched” experiment, facilitating efficient filtering and comparative analysis.

For the on-orbit dataset, the recorded fields are organized into six thematic sections:

### Sample profile

This section provides the fundamental identifiers for each experiment, including the sample name and sample number. These fields serve as unique keys for data tracking, cross-referencing, and linkage to raw measurements and metadata, forming the structural backbone of the dataset.

### Physical properties

This section documents the compositional and geometric characteristics of the samples, including material type, elemental species and their atomic percentages, as well as sample mass and diameter recorded before and after the experiment. These parameters enable quantitative analysis of mass loss and morphological changes, which are essential for evaluating evaporation behavior, geometric stability, and for deriving melt density.

### Chemical properties

This section captures qualitative descriptors of chemical behavior under high-temperature and vacuum conditions, including volatility, toxicity, corrosiveness, and stability. These attributes help assess material safety, chemical robustness, and environmental compatibility.

### Electromagnetic properties

This section reflects the sample’s interaction with electrostatic and magnetic fields, including charge response, magnetic characteristics, and electrical conductivity. These parameters influence levitation controllability, charging efficiency, and field-induced stability, and are particularly relevant for ensuring reliable sample manipulation during containerless processing.

### Thermophysical properties

As the core of the dataset, this section records key melt-state parameters such as melting point, density, viscosity, surface tension, specific heat capacity, thermal conductivity, thermal expansion coefficient, and liquidus temperature. Several properties are obtained through non-contact optical diagnostics and oscillation-based inversion methods. Some entries are currently incomplete due to limitations in image quality or pending post-processing and will be progressively updated.

### Microstructural properties

This section describes the post-solidification structural features of the samples, including crystal structure, phase composition, and the presence of macroscopic defects. These fields are currently unpopulated, as detailed analysis awaits the return of selected on-orbit samples, this information will enable correlation between thermophysical behavior and final microstructure, providing a more complete understanding of material evolution under microgravity.

Please note that this dataset does not include raw thermal imaging sequences, infrared temperature readings, or voltage time-series files. These raw data are archived by the corresponding research teams and may be used for future validation and physical modeling. Additionally, several on-orbit samples have not yet returned to Earth. Their structural and post-experiment data will be incrementally integrated as the mission progresses, making this dataset a continuously updated resource.

For the ground-matched dataset, the recorded fields are organized into four major sections:

### Sample information

This section contains the core descriptive metadata for each sample, including sample name, sample number, material type, elemental composition, emissivity/radiation ratio (used for temperature calibration), geometric diameter, and mass measurements before and after the heating process. These fields enable identification, material classification, and assessment of mass loss and morphological stability under vacuum, which are fundamental for evaluating volatility and reconstructing melt-state properties such as density.

### Physicochemical properties

This section includes qualitative evaluations of material behavior under thermal and vacuum conditions, such as volatility, laser absorption characteristics, and electrostatic charging response. These indicators help assess the heating efficiency, suspension controllability, and overall stability of each material system during high-temperature containerless processing in the ground-matched setup.

### Thermophysical properties

This section reports key thermophysical parameters of the molten samples, including melting point, density, viscosity, surface tension, and resonant frequency. These values are derived from synchronized temperature-time recordings and high-speed imaging of shape oscillations. The measurements serve as ground-reference benchmarks for interpreting microgravity results and for validating inversion-based property extraction methods.

### Microstructural properties

This section records macroscopic surface characteristics observed after sample melting and solidification, including visual features such as smoothness, cracking, or spallation. These post-processing observations help evaluate the structural integrity of the sample and provide context for assessing the reliability and consistency of the measured thermophysical properties.

Field definitions, measurement protocols, and procedural details are available in the accompanying documentation. This dataset provides a high-quality, well-structured foundation for thermophysical modeling, comparative material analysis, machine learning, and space experiment planning, serving as a valuable resource for advanced materials research and in-space manufacturing development.

## Technical Validation

### Validation of consistency in laser heating conditions

Both the on-orbit and ground-matched experiments employed programmable laser heating protocols to ensure comparability of thermal input under distinct gravitational conditions. The laser power settings, ramping strategies, and target temperatures were aligned across platforms. The sample’s thermal response was examined by comparing time–temperature profiles. For instance, as shown in Fig. 4a (ground) and Fig. 4c (on-orbit), the Ni_60_Nb_40_ sample exhibited similar heating behaviors and melting transitions, indicating that the laser output remained stable and controllable. This consistency establishes a reliable thermal environment for subsequent derivation of thermophysical properties.

### Accuracy of mass measurement and consistency of density reconstruction

Density estimation relies on precise sample mass measurements and accurate image-based volume extraction. Standardized weighing protocols were applied both in orbit and on the ground, with careful handling to minimize measurement uncertainty. Figure 4(b,d) present the temperature–density curves for the Ni_60_Nb_40_ sample obtained from ground and on-orbit experiments, respectively. Despite minor environmental variations, both datasets show consistent trends and smooth transitions. This validates the stability and reproducibility of the reconstruction algorithm across heterogeneous data sources, supporting the robustness of the overall data processing pipeline.

### Robustness of image acquisition and geometric processing

Accurate derivation of thermophysical properties such as viscosity and surface tension relies on robust image-based analysis. To mitigate potential distortion caused by microgravity-induced attitude fluctuations, a dedicated image processing workflow—comprising spherical fitting and geometric filtering—was employed to enhance the reliability of volume estimation. Results reconstructed across multiple samples and temperature ranges demonstrated consistent performance between the two experimental platforms, ensuring the validity of image-derived inputs for thermophysical modeling.

### Uncertainty and error analysis

A comprehensive assessment of measurement uncertainty was undertaken to ensure the robustness and comparability of the thermophysical property data reported in this study. The principal sources of uncertainty include the measurement precision and repeatability of the balance in sample mass determination, errors in image-based volume reconstruction arising from pixel resolution, contour extraction, and the assumption of axial symmetry, calibration accuracy and emissivity settings of the infrared pyrometer, as well as frequency resolution and damping characterization in oscillation-based determinations of surface tension and viscosity.

For representative samples with diameters of approximately 3 mm, the relative uncertainty in mass determination was within 0.1%, consistent with the ±0.01 mg balance precision and handling repeatability. An additional evaporation correction (≤0.5%) was applied, and its uncertainty was treated as a separate contribution, while uncertainties associated with image-based volume reconstruction contributed about 1.7%. Temperature measurement uncertainty, after emissivity calibration against a blackbody, was estimated at ±15 K. Oscillation analysis introduced 1.5% uncertainty in frequency determination and 9% in damping extraction, consistent with limitations reported in previous on-orbit ESL studies. Propagation of these contributions yields a combined relative uncertainty of 0.5% for density, 3% for surface tension and 10% for viscosity. These values originate from two main sources: (i) frequency determination, which contributes about 1.5% to the surface tension uncertainty; and (ii) damping characterization, which introduces about 9% to the viscosity uncertainty. The evaporation correction, though generally ≤0.5%, was also included in the uncertainty budget, avoiding bias in the density estimation. For density, the propagated error can be formally expressed as following:

8 $$\begin{array}{c}\frac{\delta \rho }{\rho }=\sqrt{{\left(\frac{\delta m}{m}\right)}^{2}+{\left(\frac{\delta V}{V}\right)}^{2}}\end{array}$$

where *δm* and *δV* denote the uncertainties in mass and volume, respectively. Uncertainties in surface tension and viscosity were propagated from the frequency resolution and damping extraction in the oscillation analysis.

These results indicate that the overall uncertainty of the present dataset is conservatively bounded within 10%, which is consistent with, and in some cases superior to, values reported for analogous containerless experiments conducted with the ISS-ELF. More importantly, the microgravity environment of the China Space Station effectively suppresses container-induced artifacts, natural convection, and sedimentation, thereby enabling more accurate contour reconstruction and more stable oscillation signals. This intrinsic advantage directly enhances both the precision and reproducibility of on-orbit measurements compared with ground-matched containerless methods and conventional container-based approaches, where systematic deviations frequently exceed 10%.

## Usage Notes

It can be used to support thermophysical property modeling under microgravity, validate numerical simulations of containerless materials processing, and develop machine learning models for property prediction, oscillation behavior analysis, and thermal response evaluation.

## Data Availability

The dataset described in this article has been deposited in Zenodo under 10.5281/zenodo.17156569.

## Code availability

The codes used to analyze the data in this study are available online (https://github.com/CSU-SPACE/CMPR_Levitation/tree/main/scripts).
