# Correction: Materials dataset from microgravity levitation experiments on the China Space Station

**DOI:** 10.1038/s41597-026-07369-y

**Published:** 2026-05-08

**Authors:** Yanan Liu, Shengyang Li, Bo Yang, Yunfei Liu, Yunziwei Deng, Jianding Yu, Xuzhi Li

**Affiliations:** 1 Technology and Engineering Center for Space Utilization, Chinese Academy of Sciences, Beijing, 100094 China; 2 Key Laboratory of Space Utilization, Chinese Academy of Sciences, Beijing, 100094 China; 3 University of Chinese Academy of Sciences, Beijing, 100049 China; 4 Shanghai Institute of Ceramics, Chinese Academy of Sciences, Shanghai, 200050 China

Correction to: *Scientific Data* 10.1038/s41597-025-06428-0, published online 16 January 2026

In the original version of this Data Descriptor, the citation for the source of the schematic shown in Figure 1 was inadvertently omitted from the figure legend and reference list. This has now been corrected in the HTML and PDF versions of the article.

